# Fluorescent Pan Traps Affect the Capture Rate of Insect Orders in Different Ways

**DOI:** 10.3390/insects10020040

**Published:** 2019-02-01

**Authors:** Mani Shrestha, Jair E. Garcia, Justin H. J. Chua, Scarlett R. Howard, Thomas Tscheulin, Alan Dorin, Anders Nielsen, Adrian G. Dyer

**Affiliations:** 1 School of Media and Communication, RMIT University, Melbourne, VIC 3001, Australia; 2 Faculty of Information Technology, Monash University, Melbourne, VIC 3800, Australia; 3 Laboratory of Biogeography and Ecology, Department of Geography, University of the Aegean University Hill, GR-81100 Mytilene, Greece; 4 Centre for Ecological and Evolutionary Synthesis (CEES), Dept. of Biosciences, University of Oslo, P.O. Box 1066 Blindern, 0316 Oslo, Norway; 5 Department of Physiology, Monash University, Melbourne, VIC 3800, Australia

**Keywords:** urban environment, pan traps, native insects, habitat fragmentation

## Abstract

To monitor and quantify the changes in pollinator communities over time, it is important to have robust survey techniques of insect populations. Pan traps allow for the assessment of the relative insect abundance in an environment and have been promoted by the Food and Agricultural Organization (FAO) as an efficient data collection methodology. It has been proposed that fluorescent pan traps are particularly useful, as it has been suggested that they capture high numbers of insects in an unbiased fashion. We use a simultaneous presentation of fluorescent and non-fluorescent pan trap colours to assess how flower-visiting insects of different orders respond to visual stimuli and reveal a significant interaction between trap fluorescence and captured insect type. In particular, Coleoptera (beetles) and Lepidoptera (butterflies and moths) were captured significantly more frequently by fluorescent traps, whilst Dipterans (flies) were captured significantly less frequently by this type of pan trap. Hymenopterans (bees and wasps) showed no significant difference in their preference for fluorescent or non-fluorescent traps. Our results reveal that the use of fluorescent pan traps may differently bias insect capture rates when compared to the typical experience of colour flower-visiting insects in natural environments. Correction factors may, therefore, be required for interpreting insect pan trap data collected with different methodologies.

## 1. Introduction

Several studies have reported that global declines in insect pollinator populations are linked to phenomena including habitat fragmentation, pathogens, invasive species, climate change, and/or the widespread use of agricultural insecticides [1,2,3,4,5,6,7,8,9]. Understanding the different contributing factors to these potential mechanisms is valuable since about 35% of food production for human consumption relies on insect pollination [1,10,11,12], with an estimated value in the range of 235–577 billion US$/year [12]. It is, thus, important to reliably quantify the relative abundance of potential flower-visiting insects in different environments to assist our understanding of changes in plant pollinator interactions, especially when considering habitat fragmentation caused by urbanization and agricultural intensification [1,5,7].

Studies and surveys of potential pollinating insects have employed pan traps (also called bowl traps) to estimate flower visitor numbers in a variety of different habitats. Pan traps have been proposed as an efficient method to collect insects from within a habitat with minimum sampling biases [13,14,15,16,17,18,19,20,21,22,23,24]. Different insect species, however, may present preferences in their perception of different colours [25,26,27,28,29,30]. For example, bees have trichromatic colour perception with ultraviolet-, blue-, and green-sensitive photoreceptors [31]. Some ants appear to perceive input from two different photoreceptor classes for colour perception [32]. Flies have four colour receptors that are further spectrally tuned with screening pigments [33,34]. Butterflies may have four or five photoreceptors that can also be spectrally tuned [33,35,36,37]. The dimensions of colour vision can dramatically influence how colour choices are made by these different animals [27,38]. To attempt to control for potential colour preference biases, previous studies have used differently coloured pan traps such as white, yellow, and blue, as perceived by human colour vision, to quantify the broad insect diversity potentially encountered in ecological settings [14,16,21,23]. These studies have typically used UV-fluorescent pan traps based on anecdotal evidence [39,40] that such stimuli collect more insects, although this factor of UV-fluorescence on insect capture rate has, only recently, been subject to formal testing [41].

Fluorescence is the phenomenon by which short wavelength radiation is absorbed by a material and re-emitted as longer wavelength radiation [42]. Materials with fluorescent properties may be of either biological [42] or non-biological origin (e.g., plastics or paints) [43]. For example, the effect is observed when we use UV-black-lights in nightclubs to produce UV-fluorescence from clothing, fluorescent highlighting pens, or Post-It notes (or sticky notes) [42]. Whilst fluorescence may enhance the intensity of a signal from a particular region of the spectrum, its effect typically results in an overall reduction of the total number of photons coming from a surface due to the conversion efficiency of the fluorescent material. Thus, to perceive and potentially benefit from fluorescence, the visual system of the receiver needs to be spectrally tuned to the wavelengths at which the fluorescence is produced [29,42]. Hence, it is reasonable to assume that insects with different visual systems may perceive fluorescent stimuli differently. This could induce sampling bias when using differently coloured pan traps, including the use of fluorescence, to attract insects.

Here, we address the question of whether pan traps displaying fluorescent properties may capture higher numbers of flower-visiting insects than non-fluorescent pan traps. Since Araneae (spiders) were also collected using our method and there is some evidence that spiders can visit flowers to prey on insect pollinators [44,45] or collect nectar [46], and that they are therefore potentially part of an extended pollination network, we also report the capture rate of these arthropods. Further, Orthopterans (crickets) are also included in our current analysis as these insects are pollinators of some flowering plants [47]. In our approach, we used typical colours employed previously for surveying insect populations for ecological studies [14,16,18,21]. We aim to test whether fluorescent pan traps catch more or less individuals of different orders of insects in comparison to non-fluorescent traps to inform us about the most effective way to survey potential insect flower visitors while minimising sampling bias.

## 2. Materials and Methods

### 2.1. Study Area

**Figure 1:**
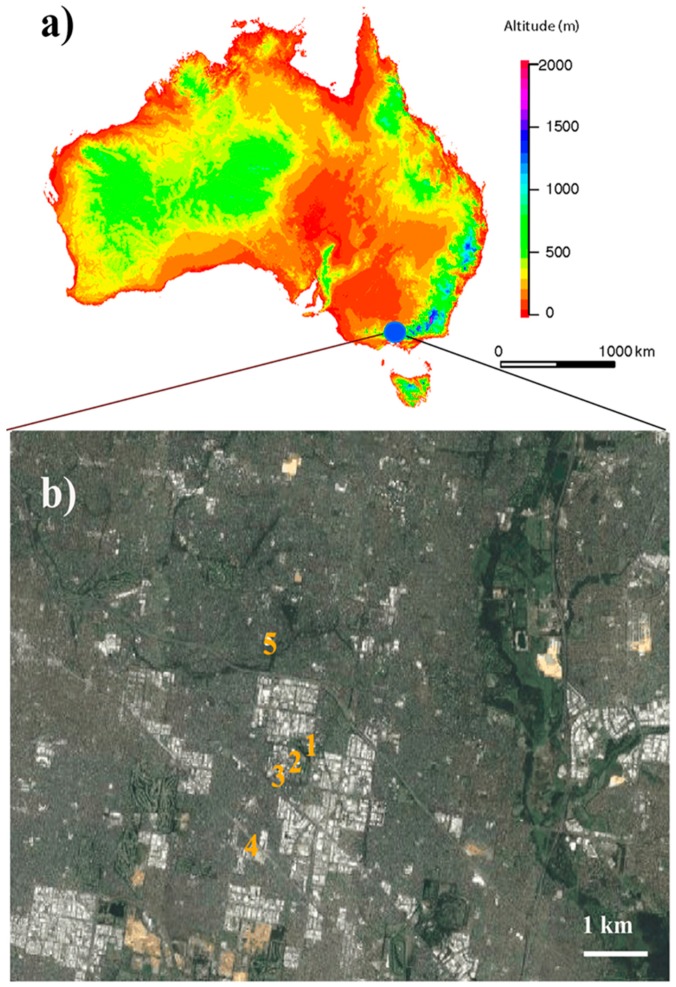
A map of the study area: (**a**) Map of Australia showing the geographical location of the study sites (blue solid circle) and (**b**) the numbers on the map show the sampling locations at Monash University, Clayton Campus and its surroundings of Melbourne, Australia. The map was prepared in R version 3.5.1 using packages “maps”, “dismo”, and “raster” [48] (R core Team 2018).

This study was conducted within the grounds of Monash University’s Clayton campus in Melbourne, Australia. The university grounds include large areas of remnant native bushland, as well as extensive gardens providing abundant resources to flower-visiting insects. The grounds are located in the temperate zone (37°53′ S–37°55′ S, 145°06′ E–145°08′ E) (Figure 1). In this study, we established five study sites and sampled insects during the Australian summer (January to May 2016) with a temperature range from 17–42 °C. Detailed temperatures for specific dates are available in Table S1.

### 2.2. Data Collection

**Figure 2:**
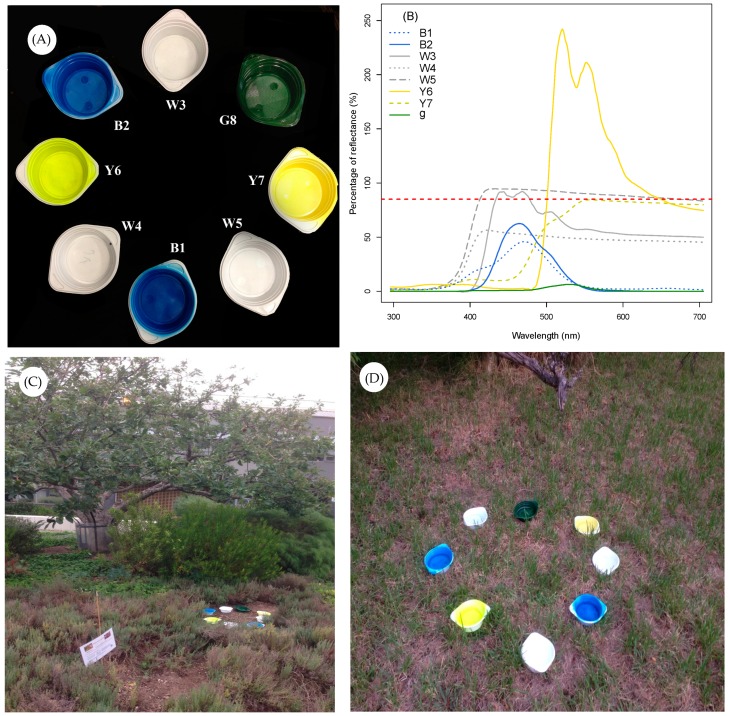
The pan trap experiment: (**A**) The pan trap bowls with eight different human perceived “colours”, (**B**) the percentage of reflected radiation plotted against wavelength for each pan trap “colour” type (See Table 1 for details and the different treatments involved for each pan trap type). The red dotted line shows the 90% threshold for categorisation as fluorescent or non-fluorescent stimuli, (**C**,**D**) the pan traps in the sample field settings.

We installed eight differently coloured pan traps at each site to sample the different groups of potential flower-visiting insects. Pan traps were separated by approximately 25 cm (Figure 2A) which ensured that successive traps were viewed using colour processing by free flying bees [31]. We used pan trap colours perceived as white, blue, yellow, and green to human vision (Figure 2A). As it is well established that insect colour vision is different to human colour vision, we provide details for each colour stimulus in Table 1 including their spectral reflectance (Figure 2B). Each pan trap cluster (*n* = 7 clusters of pan trap bowls at 5 sites) contained eight plastic soup bowls (ca. 500 mL max. vol., diameter 14 cm, depth 4.8 cm) coloured with the different paints (Figure 2A). The standard 500 mL polypropylene soup bowls (Pro-Pac, Vechta, Germany) used as pan traps were painted with fluorescent or non-fluorescent blue, white, and yellow spray paints (Sparvar Leuchtfarbe, Spray-Color GmbH, Merzenich, Germany) following the protocol of by Reference [21] and dried over several weeks to remove any residual paint smell. Each pan trap was subsequently filled with about 400 mL of water. A few drops of odourless, liquid dishwashing detergent were added to break the surface tension of the water to increase insect capture [21].

Both fluorescent and non-fluorescent pan traps were simultaneously arranged in a circle, and the location of each individual pan trap within the circle was randomly allocated per set-up and site.. The pan traps were placed on the ground for 48 h (Figure 2C,D) following standard procedures [14,16,21,23]. Sampling was repeated every two weeks at each site for four repetitions. We stored the collected insects temporarily in 70% ethanol and/or freezers before they were pinned for taxonomic identification.

### 2.3. Pan Trap Spectral Characterisation

We measured the reflectance spectrum of each pan trap colour with a spectrophotometer fitted with quartz optics and a PX-2 pulsed xenon UV-visible radiation source (USB 2000+, Ocean Optics, Dunedin, FL, USA) that closely matches the spectral profile of typical daylight illumination [49]. The spectrophotometer was attached to a computer running SPECTRA SUITE software 2011 (see References [50,51] for additional details of spectral recording methods and procedures). The reflectance spectra of the eight different pan trap types are shown in Figure 2B. Pan traps reflecting more than 90% of incident radiation at any point across the spectrum were categorized as fluorescent since very few artificial [43] or natural flower surfaces typically reflect radiation above this level [29,52]. Whilst fluorescence may work in a variety of ways and produce weak changes in colour signalling [42], we use this definition in the current study to understand what pan trap features might influence the choices of insects.

### 2.4. Insect Identification

We identified all the collected insect specimens to the order level and some specimens to the genus level, using established protocols [53,54,55,56,57,58].

### 2.5. Data Analyses

The data were recorded during the Australian summer to autumn 2016. The sampling periods spanned 48 h.

We arranged the data in a 2 × 2 contingency table to test for a potential interaction between pan trap type, i.e., fluorescent or non-fluorescent, and the order of the insects captured using a Pearson chi-square test for independence. As part of the analyses we also calculated the standardised residuals for each entry of the contingency table [59]. All analyses were performed using the package “gmodels” [60] in the R programming language version 3.4.1 [61].

## 3. Results

**Figure 3:**
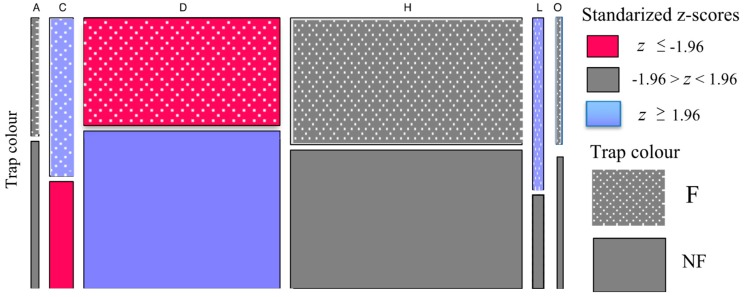
The mosaic plot shows the frequency of captures of the different insect types/order (coded with the following alphabetic letters for each group. H: Hymenoptera, D: Diptera, L: Lepidoptera, C: Coleoptera, O: Orthoptera, and A: Araneae. See Table 2 for details) for pan trap types classified as either fluorescent or non-fluorescent. The box width represents the proportion of captures for each insect order, whilst the box height is an indicator of the proportions of capture by the fluorescent pan traps (upper row dashed pattern) and the non-fluorescent traps (lower row solid pattern). The colour indicates the *z*-values for the respective standardized residuals (Table 2): blue indicates a significant preference for stimuli, red indicates that the stimuli collected significantly less individuals, and grey indicates the capture rate was not significantly different to the chance expectation (null condition). F = Fluorescent, NF = Non-Fluorescent.

When we considered the main research question, we found a significant interaction between the type of trap, either fluorescent or non-fluorescent, and the order of insect captured (χ^2^ = 27.374, d.f. = 5, *p < 0.001*) (Figure 3). The main analysis was then followed by a residual analysis to identify those insect orders presenting significantly more or less captures than what is expected by chance (Table 2). This analysis revealed that Coleopterans (beetles) and Lepidopterans (butterflies or moths) were captured more frequently on fluorescent pan traps, whilst Dipterans (flies) were captured significantly less frequently in this type of pan trap (Figure 3, Table 2). Results are graphically summarised in Figure 3.

## 4. Discussion

Pan traps are a conventional way of assessing insect–flower visitor distributions [14,15,16,17,18,19,20,21,22,23,24]. Several studies advocate the use of fluorescent stimuli due to the assumed higher rates of insect captures [14,17,21,62]. Although pan trapping with non-fluorescent traps has been used in several studies [16,41], it has rarely been considered whether the type of pan trap may bias the data collection of different insect orders due to the differences in colour processing among groups. We employed a combination of fluorescent and non-fluorescent pan trap stimuli and found that Hymenopteran insects have no significant preference for either the fluorescent or non-florescent pan traps. Other insect orders such as Coleoptera and Lepidoptera do show a preference for fluorescent pan traps (Figure 3, Table 2). In contrast, Dipterans (flies) demonstrated a preference for non-fluorescent stimuli. Our pan traps also collected some spiders (Araneae) and Orthopterans, although in relatively low numbers (Figure 3). Whilst these orders may have been an incidental by-catch, especially Orthoptera that may jump into the pan traps, these data were included in analyses as there is some evidence that Araneae and Orthoptera might participate in, or affect, pollination networks [46,47]. Neither of these orders showed any significant preference for pan traps. The evidence that Hymenopteran insects did not show a preference fits with the established literature that honeybees do not process stimulus intensity differences as a dimension of colour perception when making colour choices [63,64,65,66,67].

Currently, relatively little is known about the colour processing mechanisms of beetles, butterflies, and flies, but the spectral tuning of vision in insects of these orders is known [33,34,35,36,37] and might facilitate a capacity to process fluorescent signals [42]. Our data do suggest that such a possibility is worth exploring in detail with individual species from these insect orders. Such testing would also be of value with model bee species to validate whether indeed their visual system is insensitive to fluorescent signals as suggested by the current results.

**Figure 4:**
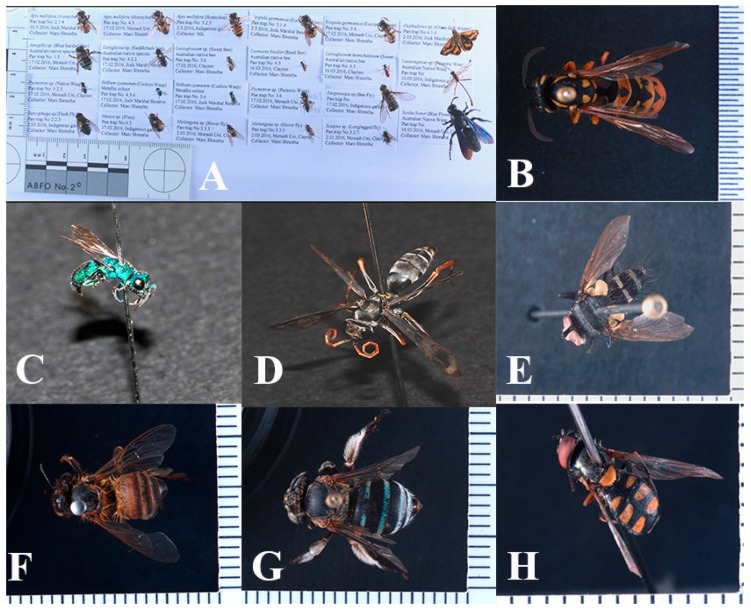
Sample insects captured by the pan traps: (**A**) Array of pinned insect samples, (**B**) European wasp (*Vespula germanica*), (**C**) cuckoo wasp (*Stibum cyanurum*), (**D**) male winged ant (*Myrmecia urens*), (**E**) long-legged fly (*Sciapus* sp.), (**F**) honey bee (*Apis mellifera*), (**G**) blue-banded bee (*Amegilla* sp.), and (**H**) hoverfly (*Melagyna* sp.). Images^©^ Copyright M.S. and J.H.C.H.

**Figure 5:**
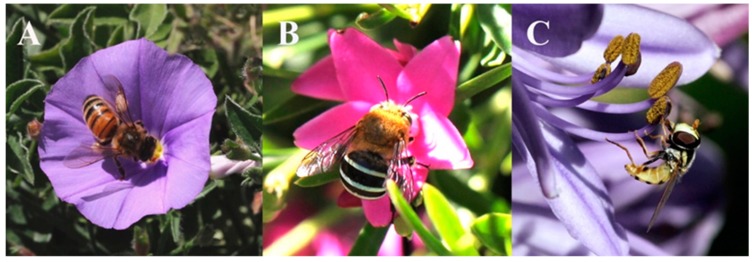
Colour photographs of flower visiting insects taken nearby the pan trap locations: (**A**) honey bee (*Apis mellifera*), (**B**) blue-banded bee (*Amegilla* sp.), (**C**) hoverfly (*Melagyna* sp.). Images Copyright^©^ M.S.

To enable efficient censuses of insect pollinators in different environments, it is important to have a robust data collection method, and the use of fluorescent pan traps has been proposed to result in higher insect capture rate [14,17,21,62]. Our observations of nearby insects visiting flowering plants confirmed that many insects captured by our pan traps were also visiting flowers in the nearby plant communities and so may be potential pollinators (Figure 4 and Figure 5) (personal observations by M.S., A.D., and A.G.D.).

The insects we captured included trichromatic native bees and introduced honeybees [68,69], hoverflies that are thought to have a four-colour visual system [34,70,71], native wasps that could potentially be trichromatic or tetrachromatic [68,69], and beetles that currently have a poorly understood colour visual system [72,73,74]. Our data on insect capture rates with either fluorescent or non-fluorescent stimuli shows that the choice of respective stimuli may result in a biased distribution (Figure 3) of the relative abundances of different pollinator groups [29], although true bias is difficult to assess in outdoor experiments with free-flying insects where overall densities are typically unknown. Future work should dissect how the spectral profiles of coloured pan trap stimuli (Figure 2B) may be perceived by different insects and how the observed preferences might influence which flower colours are pollinated [28,29,30,75,76,77]. Corrections could then be estimated from the relative ratio of fluorescent and non-fluorescent capture rates as those shown in Figure 3, although preference effects may potentially vary between species within the insect orders (Tables S1 and S2) and so corrections would benefit longer term through validation testing with individual species. We acknowledge this is very difficult: so far, colour preference testing has been successfully performed with very few species [26,27,28,29,30,31,32,75,76,77].

## 5. Conclusions

We tested if fluorescent or non-fluorescent pan trap colours captured potential flower-visiting insects in a way that might be biased due to differences in how particular insect orders may process spectral information. Whilst for Hymenopteran species there was no significant difference in the frequency of individuals caught, flower-visiting flies were preferentially captured in non-fluorescent pan traps. In contrast, fluorescent pan traps captured significantly higher rates of beetles and Lepidopterans than non-fluorescent traps, suggesting that a fundamental difference in spectral processing may have influenced the insect capture by a particular pan trap. We, thus, suggest that to survey insect populations, care in interpretation is required in the selection of pan traps colours and that corrections should be considered when conducting meta-analyses on studies with different pan trap colours.

## Figures and Tables

**Table 1:** List of the pan trap colours used in our data collection. The pan trap group (last column) in the table is categorized based on the spectral reflectance properties of each pan trap (Figure 2B).

Pan Trap Description				
Stimuli	Treatments Involved	Paint	Colour (Human Perception)	Pan Trap Group
B1	Blue paint	Blue	Blue	Non-Fluorescent
B2	Blue UV reflectance reduced	Blue “UV” fluorescent	Blue	Non-Fluorescent
W3	White UV reflectance reduced	White “UV” fluorescent	White	Fluorescent
W4	White paint	White	White	Fluorescent
W5	White	White bowl without paint	White	Non-Fluorescent
Y6	Yellow UV reflectance reduced	Yellow “UV” fluorescent	Yellow	Fluorescent
Y7	Yellow paint	Yellow	Yellow	Non-Fluorescent
g8	Green paint	Green	Green	Non-Fluorescent

**Table 2:** Summary of the *z*-scores and *p*-values for each insect order provided in Figure 3 for fluorescent and non-fluorescent pan traps. * indicates significant *p*-values at α = 0.05. *z*-scores < 0 indicate a lower frequency of choices than those expected by chance. *z*-scores ≥ 0 indicate a frequency of choices higher than expected by chance.

Insect Order	Pan Trap Type	
	Fluorescent Pan Traps	Non-Fluorescent Pan Traps
	z (*p*-Value)	z (*p*-Value)
Hymenoptera	0.981 (0.327)	−0.900 (0.368)
Diptera	−2.285 (0.022) *	2.097 (0.036) *
Lepidoptera	2.007 (0.045) *	−1.842 (0.065)
Coleoptera	2.151 (0.031) *	−1.973 (0.048) *
Orthoptera	0.107 (0.915)	−0.098 (0.922)
Araneae	−0.112 (0.911)	0.103 (0.918)

## Supplementary Materials

The following are available online at http://www.mdpi.com/2075-4450/10/2/40/s1, Table S1: Insect capture using pan traps, Table S2: A complete list of insect species sampled in this study, with numbers of individuals collected with pan traps (PT).
