# Supplementary material for: Fluorescent Pan Traps Affect the Capture Rate of Insect Orders in Different Ways
Source: Insects. 2019 Feb 1;10(2):40. doi: 10.3390/insects10020040 (PMC6410105; doi:10.3390/insects10020040)
Supplement: Supplementary file 1 [file insects-10-00040-s001.pdf]

insects

MDPI

Article

Fluorescent Pan Traps Affect the Capture Rate of Insect Orders in Different Ways

Mani Shrestha[1,2,*], Jair E. Garcia [1], Justin H. J. Chua [1], Scarlett R. Howard [1], Thomas Tscheulin [3], Alan Dorin [2], Anders Nielsen [4] and Adrian G. Dyer [1,5]

1 School of Media and Communication, RMIT University, Melbourne, VIC 3001, Australia jair.garcia@rmit.edu.au (J.E.G.); chua.heng.jie.justin@gmail.com (J.H.J.C.); scarlett.howard@rmit.edu.au (S.R.H.); adrian.dyer@rmit.edu.au (A.G.D.)
2 Faculty of Information Technology, Monash University, Melbourne, VIC 3800, Australia; alan.dorin@monash.edu
3 Laboratory of Biogeography and Ecology Department of Geography University of the Aegean University Hill, GR-81100 Mytilene, Greece; t.tscheulin@geo.aegean.gr
4 Centre for Ecological and Evolutionary Synthesis (CEES) Dept. of Biosciences, University of Oslo P.O. Box 1066 Blindern, 0316 Oslo, Norway; anders.nielsen@ibv.uio.no
5 Department of Physiology, Monash University, Melbourne, VIC, 3800, Australia
* Correspondence: sh.mani@gmail.com

Table S1. The number of insects captured using pan traps (PT) at each sampling site during various dates in Australian summer (January to May 2016). The pan traps colours are indicated by the following codes: B1 = blue-UV, B2 = blue-non-UV, W3 = white fluorescent, W4 = white UV, W5 = white UV, Y6 = yellow fluorescent, Y7 = yellow-non-fluorescent, g8 = green. In Figure 1 of the main manuscript, 1 = JMR (A–C), 2 = Indigenous garden, 3 = Monash University, 4 = Clayton, and 5 = Waverly Road.

Study Site(s)	Date(s)	B1	B2	W3	W4	W5	Y6	Y7	g8	Total
MONASH UNI	15–17 Feb 2016	1	2	5	4	3	7	10	0	32
Indigenous garden	15–17 Feb 2016	8	14	16	12	13	16	6	0	85
JMR.A	15–17 Feb 2016	3	5	10	6	10	12	7	0	53
JMR.B	15–17 Feb 2016	4	4	11	8	10	17	11	0	65
JMR.C	15–17 Feb 2016	0	8	13	14	17	5	12	0	69
Waverly Road	15–17 Feb 2016	10	11	5	12	3	17	5	0	63
Clayton	15–17 Feb 2016	15	14	8	11	10	12	6	0	76
MONASH UNI	29 Feb–2 Mar 2016	3	13	4	5	4	10	7	4	50
Indigenous garden	29 Feb–2 Mar 2016	16	12	14	7	13	17	13	8	100
JMR.A	29 Feb–2 Mar 2016	7	13	17	9	23	20	19	11	119
JMR.B	29 Feb–2 Mar 2016	12	6	14	25	17	18	17	7	116
JMR.C	29 Feb–2 Mar 2016	7	35	23	23	15	29	12	2	146
Waverly Road	29 Feb–2 Mar 2016	5	2	10	7	3	7	0	10	44
Clayton	29 Feb–2 Mar 2016	4	2	3	1	4	2	0	0	16
MONASH UNI	15–17 Mar 2016	2	3	6	7	11	12	9	2	52
Indigenous garden	15–17 Mar 2016	12	21	18	15	35	27	6	8	142
JMR.A	15–17 Mar 2016	3	7	15	2	5	15	13	15	75
JMR.B	15–17 Mar 2016	16	9	15	16	12	17	22	8	115
JMR.C	15–17 Mar 2016	10	13	19	15	21	10	15	6	109
Waverly Road	15–17Mar16	2	3	8	6	3	14	7	4	47
Clayton	15–17 Mar 2016	6	8	4	8	6	14	3	1	50
	Total	145	205	238	213	238	297	200	86	1624

Table S2. Complete list of the insect groups sampled in this study, including the total number of individuals collected by each pan trap colour (see details of the pan traps codes in Table S1 and in the main manuscript).

Species Order/PT	B1	B2	W3	W5	W4	Y6	Y7	g8	Total
**Hymenoptera**									
Ants	39	69	76	68	47	47	28	73	447
Honey Bee	1	3	3	2	2	7	1	10	29
Native Bee	10	28	17	15	13	40	0	19	142
European wasp	1	1	3	3	5	2	0	1	16
Native wasp	6	17	24	26	13	27	1	27	141
**Diptera**									
Hoverflies	0	0	0	0	8	10	0	0	18
Flies	26	25	37	50	67	76	9	35	325
Swarm fly	8	6	17	36	15	26	26	8	142
Fruit fly	2	0	1	3	3	1	0	1	11
Drosophila	1	0	2	0	1	2	0	1	7
Tiny green flies	9	3	4	8	9	20	5	10	68
Tiny midges	7	4	10	7	7	9	0	23	67
Mosquito	2	0	3	3	4	5	9	2	28
**Lepidoptera**									
Butterflies	3	3	5	2	2	9	0	6	30
Moths	1	1	0	0	0	6	0	0	8
**Coleoptera**									
Beetles	20	31	1	0	0	2	0	17	71
Weevils	3	2	2	0	1	0	4	0	12
**Orthoptera**									
Grasshopper	1	3	0	0	1	1	2	1	9
Cricket	2	0	1	1	0	0	0	1	5
**Araneae**									
Spider	2	4	6	3	1	3	1	3	23
**Unknown Insects**	2	5	1	11	1	5	0	0	25
Total	146	205	213	238	200	298	86	238	1624

© 2019 by the authors. Licensee MDPI, Basel, Switzerland. This article is an open access article distributed under the terms and conditions of the Creative Commons Attribution (CC BY) license (http://creativecommons.org/licenses/by/4.0/).
